# Equality and social determinants of spatial accessibility, availability, and affordability to primary health care in Hong Kong, a descriptive study from the perspective of spatial analysis

**DOI:** 10.1186/s12913-022-08760-2

**Published:** 2022-11-17

**Authors:** Xuechen Xiong, Victor Jing Li, Bo Huang, Zhaohua Huo

**Affiliations:** 1 Institute of Future Cities, The Chinese University of Hong Kong, Hong Kong, China; 2 School of Public Health, Fudan University, Shanghai, China; 3 Department of Geography and Resource Management, The Chinese University of Hong Kong, Hong Kong, China; 4 JC School of Public Health and Primary Care, The Chinese University of Hong Kong, Hong Kong, China

**Keywords:** Primary health care, Accessibility, Availability, Affordability, Spatial regression

## Abstract

**Objective:**

Primary health care (PHC) is widely perceived to be the backbone of health care systems. Since the outbreak of COVID-19, PHC has not only provided primary medical services, but also served as a grassroots network for public health. Our research explored the accessibility, availability, and affordability of primary health care from a spatial perspective, to understand the social determinants affecting access to it in Hong Kong.

**Method:**

This constitutes a descriptive study from the perspective of spatial analysis. The nearest neighbor method was used to measure the geographic accessibility of PHC based on the road network. The 2SFCA method was used to measure spatial availability and affordability to primary health care, while the SARAR model, Spatial Error model, and Spatial Lag model were then constructed to explain potential factors influencing accessibility and availability of PHC.

**Results:**

In terms of accessibility, 95% of residents in Hong Kong can reach a PHC institution within 15 minutes; in terms of availability, 83% of residents can receive PHC service within a month; while in terms of affordability, only 32% of residents can afford PHC services with the support of medical insurance and medical voucher. In Hong Kong, education status and household income show a significant impact on accessibility and availability of PHC. Regions with higher concentrations of residents with post-secondary education receive more PHC resources, while regions with higher concentrations of high-income households show poorer accessibility and poorer availability to PHC.

**Conclusion:**

The good accessibility and availability of primary health care reflects that the network layout of existing PHC systems in Hong Kong is reasonable and can meet the needs of most residents. No serious gap between social groups further shows equality in resource allocation of PHC in Hong Kong. However, affordability of PHC is not ideal. Indeed, narrowing the gap between availability and affordability is key to fully utilizing the capacity of the PHC system in Hong Kong. The private sector plays an important role in this, but the low coverage of medical insurance in outpatient services exacerbates the crowding of public PHC and underutilization of private PHC. We suggest diverting patients from public to private institutions through medical insurance, medical vouchers, or other ways, to relieve the pressure on the public health system and make full use of existing primary health care in Hong Kong.

## Background

### Primary health care in Hong Kong

Ideally, primary health care (PHC) should bring healthcare as close as possible to where people live and work, and constitutes the first element of an ongoing healthcare process. PHC is widely perceived to be the backbone of a national health care system [1, 2], with evidence demonstrating that higher quality primary care is associated with better health quality outcomes, higher patient satisfaction, and lower health-related inequity at lower cost [3–6]. As such, many countries are strengthening community-based primary care, focusing on prevention and quality improvement in disease management [7–9].

In Hong Kong, family doctors are the main primary care service providers to residents [10]. Strengthening the role of primary care is a key element of the ongoing reform strategy in Hong Kong [11, 12]. In 2008, the Government set out a comprehensive package of proposals for reforming the healthcare system, including enhancing primary care development. A year later, in 2009, it published the "Primary Care Development in Hong Kong: Strategy Document", setting out the major strategies and pathways of action for improving the delivery of primary care services. Then in 2017, Hong Kong set up the District Health Centre (DHC) as a pilot to strengthen medical-social collaboration and public-private partnership [12]. Whilst this is a worthy aspiration, it is no easy task to achieve because the current primary care system in Hong Kong remains provider-dominated and fragmented [13–15]. In Hong Kong, 70% of primary care is provided by the private sector, while the other 30% is mainly provided by 73 government-run general outpatient clinics (GOPCs) [16–18]. GOPCs provide service to all the population but target the poor and the elderly. Primary care is more frequently managed privately, due to greater accessibility compared with public care [19]. However, elderly people, particularly those with lower incomes, tend to go to the publicly funded general out-patient clinics for their primary care, as the majority cannot afford private healthcare service [20]. Thus, affordability is said to be an impediment to receiving primary care in Hong Kong. Introduced in 2009, the Elderly Health Care Voucher Scheme provides an annual allowance in the form of vouchers [21] for citizens aged over 65 years to use on private outpatient services, which restructured health financial equitably for elderly patients to seek private providers [22, 23].

### Spatial access of primary health care

Accessibility of healthcare is a broad and flexible concept. Scholars have proposed a classic understanding of “access”, defining the measurement dimensions of health accessibility as availability, accessibility, accommodation, affordability, and acceptability [24]. But the dimensions of access to healthcare are not always applied as they were originally conceptualized, and have been modified variously in subsequent studies [25–28]. Access to primary health care (PHC) is considered a fundamental right and an important facilitator of overall population health [29, 30]. Ensuring equal access to primary care for those in equal need has long been of concern to public health policy makers, service providers, researchers, and consumers alike [31–33]. Spatial access to healthcare refers to the geographic distribution of physicians and patients. It constitutes a measure of healthcare delivery and encompasses both accessibility and availability: travel costs paid by patients as well as the adequacy of the physician supply at the point of care [34–39]. This article focuses on the measurement of potential spatial accessibility and availability, exploring spatial affordability, while ignoring aspatial considerations like acceptability or accommodation.

An accurate assessment of PHC is essential to understand and address existing inequalities, thus narrowing the gaps in access to PHC [40, 41]. Methods measuring spatial access can be roughly classified into the following categories: provider-to-population ratios (PPR); distance to nearest provider; and gravity models [26]. Many new techniques have been introduced based on the gravity model, of which the Two-Step Floating Catchment Area (2SFCA) method has gained the most traction [42]. Based on this, several modifications and extensions have been suggested [43–46]. In this paper, distance to nearest provider and 2SFCA methods were used to assess the accessibility, availability, and affordability of PHC in Hong Kong.

### Factors influencing access of primary health care

It is necessary to analyze the factors that influence spatial access to PHC, as they are essential for conditioning equitable access to strengthen Hong Kong’s primary care system [47]. Linear regression analysis is used to develop a regression model and test whether independent variables influence the dependent variable. Ordinary least squares (OLS) is one of the most frequently used methods to estimate the parameters of linear regression models [48], with several assumptions that must be fulfilled. In the context of spatial data, OLS is not appropriate, because spatial dependence is one of the main consequences of using geographical data [49]. Spatial dependence occurs when the accessibility present in a particular suburb is determined both by its own characteristics as well as by the characteristics of nearby properties. Spatial regression models can thus deal with the spatial dependence issue. Many researchers have applied spatial regression models to analyze social problems [50–52]. In this paper, we use spatial regression model to analyze the social factors affecting access to primary health care.

In existing literature, many studies have analyzed or evaluated the spatial accessibility or availability of PHC services [53–57]. However, few articles incorporate the other aspatial factors, like affordability into the measurement of spatial access. Additionally, researches focusing on Hong Kong mainly discuss the delivery system of primary care [58, 59] and reflect on social factors influencing the utilization of primary care [19, 59]. By contrast, few studies provide a comprehensive map of spatial access to primary health care in Hong Kong or analyze the factors affecting potential access to PHC. This study takes Hong Kong as the study area, integrating accessibility, availability, and affordability of primary health care in the perspective of spatial view, modeling the equality of accessing PHC, using a spatial regression model to better understand the social determinants affecting access to it in Hong Kong.

## Methods

In this paper, the accessibility, availability, and affordability of PHC in Hong Kong is descripted through the nearest neighbor and 2SFCA methods, respectively. The following sections provide details of the dataset used in this study as well as the measuring methods employed.

### Datasets

#### Calculating units

For town planning purpose, the whole territory of Hong Kong is divided into smaller units of Large Street Block Groups (LSBGs), Street Blocks (SBs) in urban areas or Village Clusters (VCs) in rural areas. While maintaining regard for data precision and the privacy of individual respondents, a Street Block with a small population has been merged with adjacent SBs/VCs to form Large Street Block Groups in released statistics [60]. As a result, we observe 1,622 LSBGs in the whole territory of Hong Kong. In this paper, we conducted regression analysis and visualizations all using the geographic unit of LSBGs.

#### Population data

Hong Kong has approximately 7.4 million people. Population density is about 6,801 per km^2^ for the whole territory. Detailed population data from Street Blocks (SBs) or Village Clusters (VCs) was collected from Hong Kong 2016 Population By-census [61]. Besides the population information, it provides benchmark statistics on the socio-economic characteristics of the Hong Kong population residing in LSBGs, which are the explanatory variables in the regression model.

#### Supply of PHC

The Hospital Authority (HA) is a statutory body providing public hospitals and related services to the citizens of Hong Kong. It offers primary health care services to patients through 73 general out-patient clinics. The detailed list of the general out-patient clinic can be collected from the official website [62]. Moreover, thousands of clinics managed by private sector provide primary medical services. The private clinics are registered and can be searched from the official website [63]. For these private clinics, only general private clinics, including Traditional Chinese Medicine clinics were recruited in analysis. In total, 73 general outpatient clinics managed by public sector and around 5,000 general outpatient clinics managed by private sector were set as the provider of primary health care for residents in Hong Kong.

#### Demand for PHC

We referenced the public report of Thematic Household Survey (THS) of Hong Kong, which investigated the general health system of Hong Kong with residents’ health status and utilization of health services [64]. Based on the population information in LSBGs combined with the utilization of medical service from THS report for each age and gender group, the demands for PHC visits were estimated over a month. Additionally, the THS provided information on health insurance covering doctor consultation. Based on the population information in LSBGs combined with the medical insurance coverage from THS report for each age group, the distribution of residents with medical insurance covering doctoral consultation were estimated.

#### Network data

The network data was sourced from LBS (lbs.amap.com) [65]. The dataset contains spatial information and attributes, such as road network, buildings, point of interests, and provides necessary information to estimate distance between two locations. Building data are recorded by individual buildings, with attributes including the floor of the building and property of the building. According to these attributes, buildings used as residential housing can be selected as accurate geographical units in simulating population distribution.

### Measuring methods

#### Nearest neighbor method

The nearest neighbor method was used to measure geographic accessibility to PHC based on the road network. It assumed that distance is the most important factor for people to choose PHC, and patients are more inclined to choose the closest institution to obtain PHC services. The shorter the time to the nearest PHC facility, the better the accessibility of PHC is for residents. Based on the distribution PHC institutions, as well as the distribution of residential buildings in Hong Kong, the time cost to reach the nearest medical institution was calculated.

#### 2SFCA method

2SFCA method was used to measure spatial availability to PHC. Within a certain catchment, patients sometimes have multiple medical facilities to choose from. Similarly, a medical institution that serves many patients can be crowded. The nearest neighbor method does not take into the relationship between supply and demand; while the 2SFCA method, based on the gravity model, considers its supply capacity, service demand, and proximity features. The 2SFCA method conducts two searches. The first step is to set supply point j as the center, search the demand point i within the threshold range of supply point j, and calculate the supply-demand ratio within the threshold range. The second step is to search the supply points within the threshold range with demand point i as the center, and then sum the supply and demand ratios of all supply points to obtain the accessibility of demand point i, as Ai. The method can be represented as follows.

1 $${\textrm{A}}_{\textrm{i}}={\sum}_{\textrm{j}\in \left\{{\textrm{d}}_{\textrm{ij}}\leq {\textrm{d}}_0\right\}}{\textrm{R}}_{\textrm{j}}=\kern0.5em {\sum}_{\textrm{j}\in \left\{{\textrm{d}}_{\textrm{ij}}\leq {\textrm{d}}_0\right\}}\frac{{\textrm{S}}_{\textrm{j}}}{\sum_{\textrm{k}\in \left\{{\textrm{d}}_{\textrm{kj}}\leq {\textrm{d}}_0\right\}}{\textrm{D}}_{\textrm{k}}}$$

Where i represents the demand point; j is the supply point; d_ij_ represents the distance between demand point i and supply point j; R_j_ represents the ratio of the service capacity of supply point j to the demand served within the search threshold; D_k_ represents the service demand at demand point k; S_j_ represents the service capability of supply point j; Ai presents the accessibility of demand point i calculated by the 2SFCA method. If Ai <1, it means the supply of PHC cannot satisfy the demand of residents. If Ai = 1, it means the supply of PHC fit the demand of residents. If Ai >1, it means the supply of PHC is above the demand of residents.

In this paper the spatial affordability of PHC is not about the concrete financial burden for residents to receive PHC services. Rather, we calculated the affordability index based on the availability index (Ai). We assumed that residents who have medical insurance covering private outpatient service would prefer service from private PHC institutes, while residents without medical insurance covering outpatient service, will take public PHC as the first choice in process of seeking PHC. The Ai index was used here to express the affordability of PHC services for Medicare and non-Medicare residents. The difference is that the Ai index in availability is irrespective of selection preferences, while the Ai index in affordability considers health seeking choices among public clinics and private clinics.

### Spatial Regression Method

#### Variables

Spatial regression was used to analyze the impact of accessibility and availability of PHC, with explanatory variables as follows: aged 65 and above (X1); born in HK (X2); primary education (X3); post-secondary education (X4); and low household income (X5); high household income2(X6); owner-occupier households(X7); household rent to income (X8); and accessibility, availability as response variables respectively. Variance inflation factor (VIF) is used to detect whether multicollinearity exists in a regression model. All explanatory variables got the VIF score under 10 in accessibility regression model and availability regression model. The descriptive statistics of explanatory variables in the empirical analysis are showed in Table 1.

**Table 1:** Summary of variables and descriptive statistics

**Variable**	**Description**	**Mean**	**SD**
Aged 65 and over (X_1_)	Proportion of residents aged 65 and above	0.15	0.07
Born in HK (X_2_)	Proportion of residents born in HK	0.61	0.12
Primary education (X3)	Proportion of residents with primary education or below	0.17	0.09
Post-secondary education (X4)	Proportion of residents with post-secondary education	0.36	0.14
Low household income (X5)	Proportion of residents with domestic household income per month less than 10,000HK$	0.18	0.09
High household income (X6)	Proportion of residents with domestic household income per month over 40,000 HK$	0.36	0.21
Owner-occupier household (X7)	Proportion of residents who are owner-occupier households	0.55	0.25
Household rent to income (X8)	Median ratio of monthly household rent to income	0.26	0.12

### SARAR model

The SARAR (Spatial AutoRegressive with additional AutoRegressive error structure) model follows an autoregressive process, which is indicated by the presence of dependence relationship among a set of observations or spatial units [66, 67]. It is written as

2 $${\displaystyle \begin{array}{c}Y=\uprho \textrm{WY}+\textrm{X}\upbeta +\textrm{u},\\ {}\upmu =\uplambda \textrm{M}+\upvarepsilon, \kern0.5em \upvarepsilon \sim \textrm{N}\left(0,{\sigma}^2\textrm{I}\right)\end{array}}$$

Where Y is an n-by-1 vector of response variables, X is an n-by-p matrix of explanatory variables; ρ is spatial lag coefficient parameter that will determine the strength of the spatial autoregression on the response variable and |ρ| < 1; λ is a spatial error parameter that will determine the strength of the spatial autoregression on μ and |λ| < 1; μ is an n-by-1 vector of error terms assumed to have autocorrelation; β is a p-by-1 vector regression coefficients; and W, M are standardized spatial weights matrix (n×n) ( ∑_j_w_ij_ = 1, ∑_j_m_ij_ = 1 ) where n is the number of observation. Each observation of the spatial-lag variable is a weighted average of the values of the dependent variable observed for the other cross-sectional units. The matrix containing the weights is known as the spatial-weighting matrix. It is a quantification of the spatial relationships that exist among the observation location. All modelling processes were calculated by R programming using “spdep” packages. The SARAR model has two types of spatial regression model.

1. Spatial lag model (SLM). The model emerges from dependence in the observation value of dependent variable in a spatial unit and the corresponding neighboring units [68].The response variable is autoregressed on spatially lagged response variables. The SLM is obtained if λ in (2) is zero, that is $${\displaystyle \begin{array}{cc}\textrm{Y}=\uprho \textrm{WY}+\textrm{X}\upbeta +& \upvarepsilon, \upvarepsilon \sim \textrm{N}\left(0,{\sigma}^2\textrm{I}\right)\end{array}}$$
2. Spatial error model (SEM). The model emerges from the presence of spatial dependence in the error term of a spatial unit and the corresponding neighboring units. It occurs in the case that some variables influencing dependent variable value but exclude in the model correlate among spatial units. The SEM is obtained if ρ in (2) is zero, that is $${\displaystyle \begin{array}{cc}\textrm{Y}=\textrm{XB}+\upmu, \upmu =& \uplambda \end{array}}{\textrm{W}}_{\upmu}+\upvarepsilon, \upvarepsilon \sim \textrm{N}\left(0,{\sigma}^2\textrm{I}\right).$$

To identify whether there is autocorrelation in the values of the dependent variable or in its errors, a Lagrange Multiplier (LM) test and a Robust Lagrange Multiplier (Robust-LM) test were conducted. Goodness-of-fit statistics such as the R-squared, Log likelihood, AIC and BIC can be used to estimate the fitting degree of regressions.

## Results

### Accessibility to PHC

**Fig. 1:**
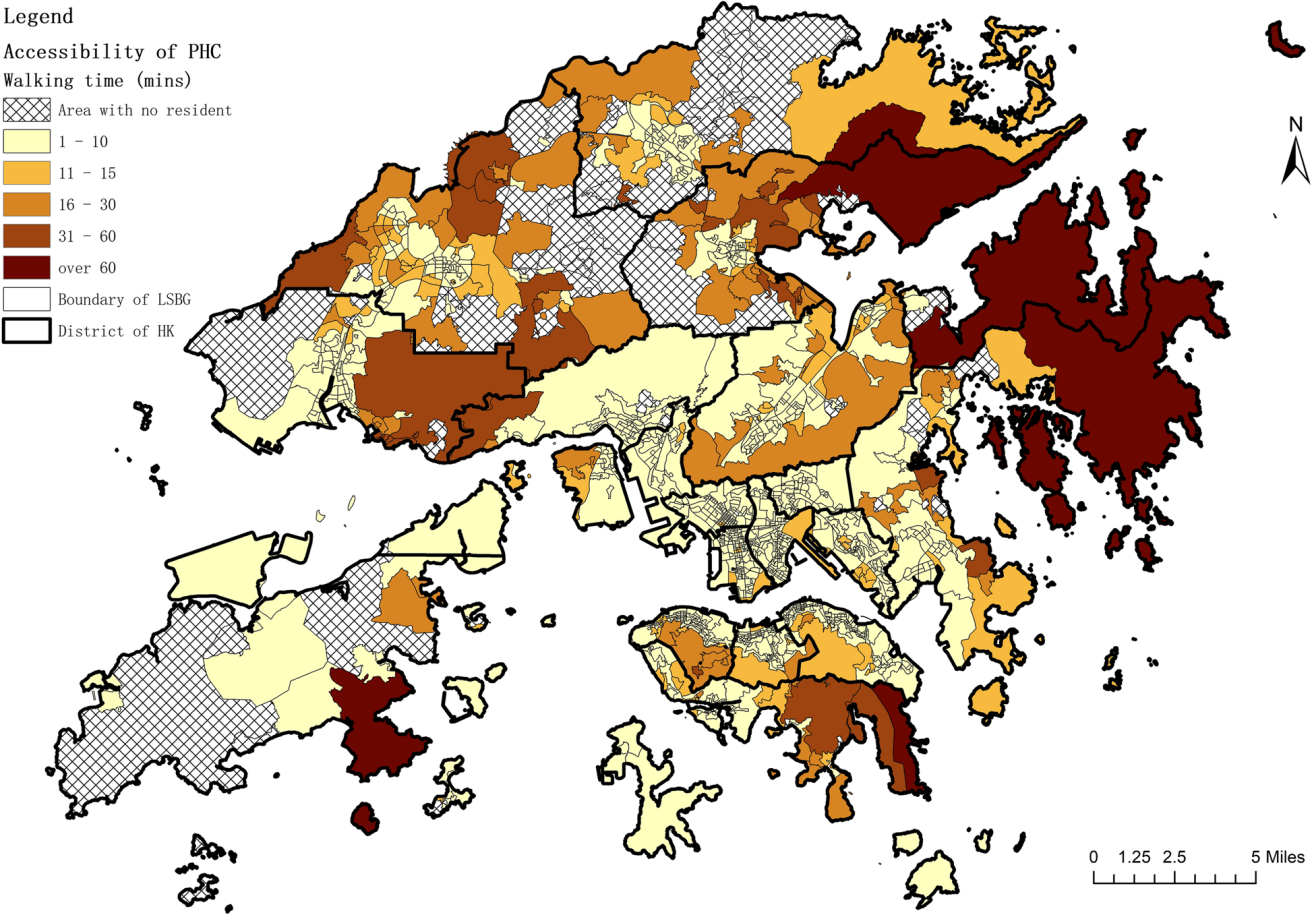
Travel time map in accessing the nearest PHC institution

**Fig. 2:**
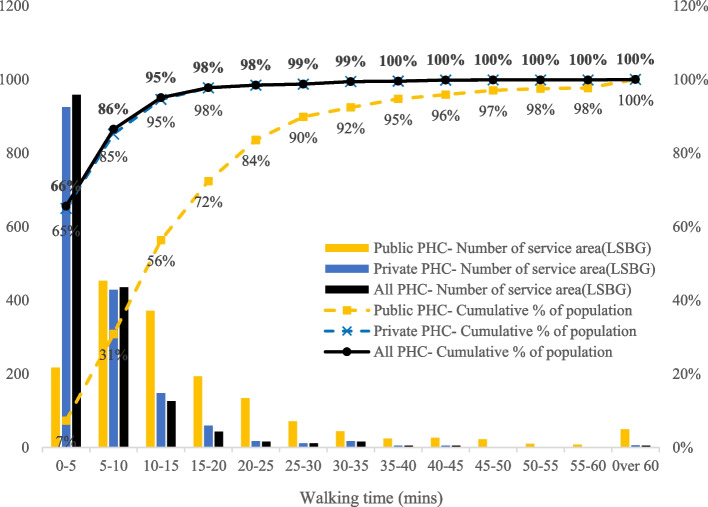
Travel time histogram in accessing the nearest PHC institution

From the analysis of time cost in accessing the nearest PHC facility, we deduced that in Hong Kong, the maximum walking time for residents to the nearest PHC facility is over 1 hour, while the average walking time is around 5.21 min (SD ±6.55min). Figure 1 shows the travel time map, while Fig. 2 shows the statistical chart of access time to public and private PHC and the coverage of the population. Among 1,622 LSBGs, over half LSBGs with 66% of residents can reach a PHC institution within 5 minutes: 435 LSBGs with 21% of residents can reach a PHC institution in 5-10 minutes; 148 LSBGs with 9% of residents can reach a PHC institution in 10-15 minutes; 70 LSBGs with 4% of residents can reach a PHC institution in 15-30 minutes; 28 LSBGs with 1% of residents can reach a PHC institution in 30-60 minutes; and 5 LSBGs with 0.1% of residents can reach a PHC institution in over 60 minutes.

In summary, 95% of residents in Hong Kong can reach a PHC institution within 15 minutes, 99% of residents can reach a PHC institution within 30 minutes, and almost all residents can reach a PHC institution within 60 minutes. There is a discrepancy between the contribution of public sector and private sector to the accessibility of PHC. For public PHC institutions, 56% of residents can reach one within 15 minutes, 90% of residents can reach one within 30 minutes, and 98% of residents can reach one within 60 minutes. For private PHC institutions, 95% of residents can reach one within 15 minutes, 99% of residents can reach one within 30 minutes, and almost all residents can reach one within 60 minutes.

### Availability of PHC

**Fig. 3:**
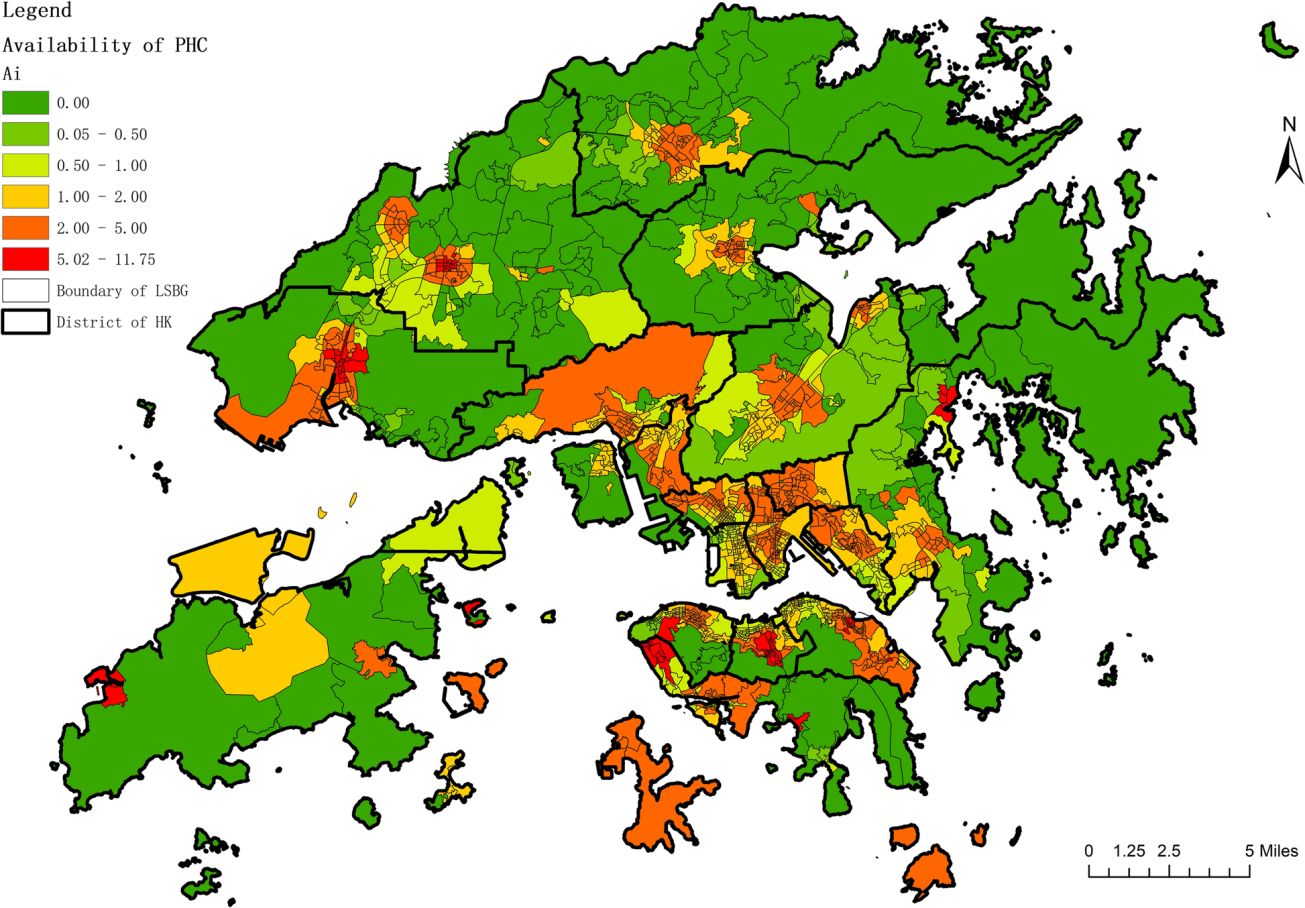
Availability map in accessing PHC

**Fig. 4:**
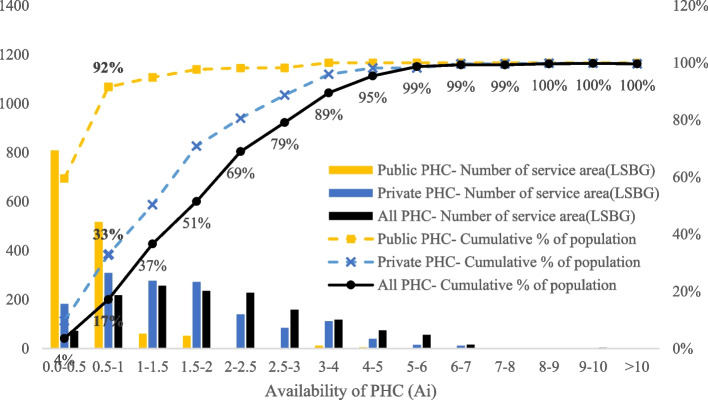
Availability histogram in accessing PHC

The 2SFCA method was used to express he availability of PHC within the threshold range of 15 minutes walking distance with the index of A_i_. From the analysis of availability, it was determined that in Hong Kong the maximum value of A_i_ is over 10, and the average is around 2.06 (SD ±1.44), as shown in Fig. 3. Figure 4 presents the statistical chart of availability to public and private PHC and the coverage of the population. It reveals that the Ai value of 72 LSBGs with 4% of residents is less than 0.5, and the Ai value of 217 LSBGs with 14% of residents is greater than 0.5 and less than 1. This means that in a month, 17% of residents cannot get PHC within the threshold range of 15 minutes walking distance. On the other hand, 491 LSBGs with 34% of residents have an Ai value between 1 and 2; 386 LSBGs with 28% of residents have an Ai value between 2 and 3; 118 LSBGs with 10% of residents have an Ai value between 3 and 4; 74 LSBGs with 6% of residents have an Ai value between 4 and 5; and 74 LSBGs with 4% of residents have an Ai value greater than 5.

In summary, 83% of residents in Hong Kong can receive PHC service within the threshold range of 15 minutes walking distance in a month (Ai>1). Among them, 48% of the residents are able to access PHC supplies that are greater than twice their needs (Ai>2); 21% of the residents to PHC supplies that are more than three times their needs (Ai>3); a 4% of the residents have access to PHC supplies that are more than five times their needs (Ai>5). There is a discrepancy between the contribution of public and private sectors to the availability of PHC. Only 8% of residents are able to receive public PHC services within the threshold range of 15 minutes walking distance, while 67% of residents can receive private PHC services.

### Affordability of PHC

We assumed that residents who have medical insurance covering private outpatient service would prefer service from private PHC institutes, while residents without medical insurance covering outpatient service, will take public PHC as their first choice in the process of seeking PHC. The Ai index was used here to express the availability of PHC services for Medicare and non-Medicare residents. For people without medical insurance seeking public PHC services, 58% are assigned an Ai value of less than 0.5; and 33% of them an Ai value greater than 0.5 and less than 1. This means that in a month, only 9% of residents who have no medical insurance can afford PHC service. For people with medical insurance seeking private PHC services, 0.2% of them have an Ai value less than 1, while 5.2% have an Ai value greater than 1 and less than 10, and 94% of them have an Ai value greater than 10. This means that in a month, almost all residents (99.8%) who have medical insurance can afford the PHC they need within the threshold range of 15 minutes walking distance.

In addition to medical insurance, the medical voucher scheme for the elderly also increases the affordability of medical products for the target population. Assuming that all Hong Kong seniors over the age of 65 are eligible for the medical voucher scheme and are willing to obtain private services to avoid crowding public services, then for people without medical voucher or insurance, 20% can afford PHC within the threshold range of 15 minutes walking distance. By contrast, for people with insurance or medical voucher, 95% can afford PHC.

**Fig. 5:**
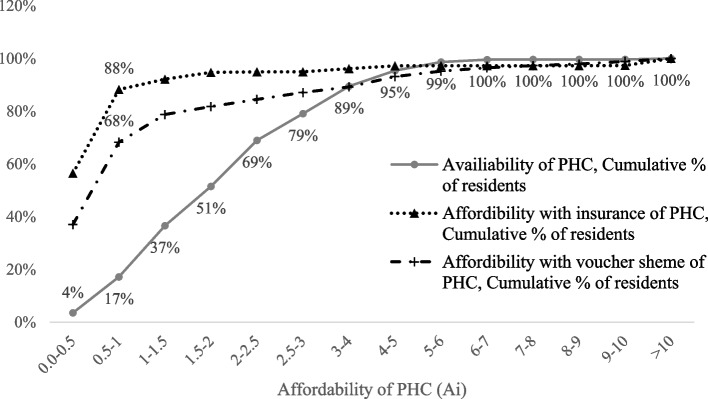
Affordability compared with accessibility in accessing PHC

In summary, with health insurance, 12% of residents in Hong Kong can afford PHC service within the threshold range of 15 minutes walking distance (Ai>1) in a month. The other 88% residents cannot afford it. With the additional support of medical vouchers, 32% residents in Hong Kong can afford PHC service within the threshold range of 15 minutes walking distance (Ai>1) in a month, while 68% residents cannot afford it. Thus, there remains a large gap between the availability and affordability of PHC in Hong Kong, even with help from medical insurance and medical vouchers for the elderly (Fig. 5).

### Social determinants

#### Social Factors influencing accessibility of PHC

The results of spatial models are similar for most of the variables used in the regression analysis of accessing time to the nearest PHC facilities. The SARAR model explains about 29% of the variation in access time. The ρ coefficient is 0.37, which indicates that if an area is surrounded by other regions, then the influence of each region can be measured by 0.37 times the dependent variable around it; a value of the spatial error parameter λ is 0.33. Significance test of model parameter estimates revealed that the variable of post-secondary education (X4), low household income (X5) and high household income (X6), and ρ coefficient had significant effect. Table 2 details the regression results.

**Table 2:** The regression results of accessibility and availability of PHC

Variable	Accessibility to PHC				Availability of PHC			
	OLS	SLM	SEM	SARAR	OLS	SLM	SEM	SARAR
Constant	6.20 **	3.47	7.66 ***	5.01 *	1.21 **	-0.1	1.19 ***	0.12
	(2.24)	(1.98)	(2.22)	(2.40)	(0.42)	(0.24)	(0.28)	(0.29)
Aged 65 and over (X_1_)	-12.13 *	-8.62 *	-3.47	-5.57	0.52	0.76	0.43	0.65
	(4.97)	(4.38)	(4.55)	(4.51)	(0.93)	(0.52)	(0.53)	(0.55)
Born in HK (X_2_)	-3.77	-2.55	-3.14	-2.88	1.57 ***	0.4	0.45	0.54
	(2.43)	(2.15)	(2.51)	(2.38)	(0.46)	(0.26)	(0.31)	(0.29)
Primary education (X3)	-1.23	-1.52	-6.23	-4.42	1.06	0.23	0.32	0.2
	(5.39)	(4.75)	(4.88)	(4.86)	(1.01)	(0.57)	(0.57)	(0.60)
Post-secondary education (X4)	-18.02 ***	-14.76 ***	-14.03 ***	-14.57 ***	1.89 **	1.18 **	0.95 *	1.06 *
	(3.64)	(3.21)	(3.48)	(3.39)	(0.68)	(0.38)	(0.41)	(0.42)
Low household income (X5)	15.76 ***	12.63 ***	12.13 ***	12.67 ***	-1.44 *	-0.28	0.03	-0.13
	(3.89)	(3.44)	(3.51)	(3.51)	(0.73)	(0.41)	(0.41)	(0.43)
High household income (X6)	21.63 ***	16.56 ***	14.18 ***	15.34 ***	-3.61 ***	-1.23 ***	-0.52	-1.01 **
	(3.23)	(2.85)	(2.95)	(2.94)	(0.61)	(0.34)	(0.34)	(0.36)
Owner-occupier household (X7)	-0.57	-0.2	0.22	0.1	0.35	0	-0.13	-0.07
	(1.14)	(1.00)	(1.02)	(1.02)	(0.21)	(0.12)	(0.12)	(0.13)
Household rent to income (X8)	1.45	0.19	-1.12	-0.72	-1.37 **	-0.19	-0.34	-0.3
	(2.38)	(2.10)	(2.23)	(2.21)	(0.45)	(0.25)	(0.26)	(0.27)
ρ		0.54 ***		0.37 *		0.79 ***		0.66 ***
		(0.02)		(0.19)		(0.01)		(0.08)
λ			0.54 ***	0.33			0.80 ***	0.33 **
			(0.02)	(0.20)			(0.01)	(0.12)
R^2	0.05	0.29	0.30	0.29	0.04	0.71	0.71	0.70
AIC		11269.51	11282.26	11233.44		4589.12	4598.45	4573.5
BIC		11339.6	11352.35	11308.92		4659.21	4668.54	4648.98
Deviance		90234.99	90876.11	89681.86		1289.34	1292.94	1350.14
Log Likelihood		-5621.76	-5628.13	-5602.72		-2281.56	-2286.22	-2272.75
LMlag		243.0***				1791.8***		
RLMlag		18.7***				51.6***		
LMerr			226.1***				1823.0***	
RLMerr			1.8				82.8	

Note: *** *p* < 0.001; ** *p* <0.01; * *p* <0.05

*PHC* Primary health care, *OLS* indicates the ordinary least squares regression method, *SLM* indicates the spatial lag model, *SEM* indicates the spatial error model; SARAR indicates the spatial autoregressive with additional autoregressive error structure model

The ratio of post-secondary education (X4) had a negative relationship with access time to the nearest PHC facility. Assuming the condition of other variables is constant, the increase in percentage of the ratio of post-secondary education by 1 unit can reduce the access time of a region by 14.57 mins. That is, the higher the ratio of residents with post-secondary education in a region, the less time cost exists to access the nearest PHC facilities. Low household income (X5) and high household income (X6) both had a positive relationship with access time to the nearest PHC facility. Assuming the condition of other variables is constant, the increase in the ratio of low-income household by 1 unit can increase the access time by 12.67 mins. Similarly, if the ratio of high-income household rose by 1 unit in a region, then the access time would increase by 15.34 mins. That is, the higher the ratio of low-income household or high-income household in a region, the higher the time cost to access the nearest PHC facilities.

#### Social Factors influencing availability of PHC

The SARAR model explains about 70% of the variation in availability of PHC in Hong Kong. The ρ coefficient is 0.66, which indicates that if an area is surrounded by other regions, then the influence of each region can be measured by 0.66 times the dependent variable around it; a value of spatial error parameter λ is 0.33. Significance test of model parameter estimates showed that the variable of post-secondary education (X4), high household income (X6), ρ coefficient and spatial error λ had significant effect. Table 2 details the regression results.

High household income (X6) exhibited a negative relationship with availability of PHC service. Assuming the condition of other variables is constant, the increase in ratio of high household income in a region by 1 unit, can reduce the availability of a region by 1.01. It showed higher concentrations of residents with high income not only had poorer accessibility to, but also poorer availability of PHC. The variable of post-secondary education (X4) had a positive relationship with availability of PHC service. Assuming the condition of other variables is constant, the increase in ratio of post-secondary education in a region by 1 unit can increase the average availability of a region by 1.06. That is, the higher the ratio of post-secondary education in a region, the better accessibility and better availability to PHC facilities in the region.

#### Access to PHC in different income groups

**Fig. 6:**
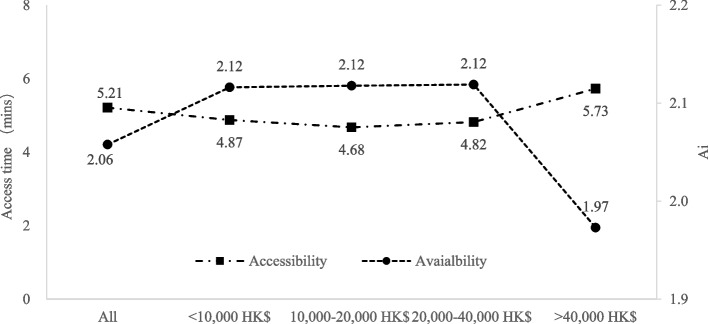
Accessibility and availability of PHC in different income groups

The regression analysis indicated that the higher the concentration of high-income households or low-income households, the poorer the accessibility of PHC facilities. While higher concentrations of residents with high income not only had poorer accessibility to, but also poorer availability of PHC. To distinguish access to PHC among people with different household income levels, we extracted the accessibility and availability of PHC among four household income groups (Fig. 6).

In terms of accessibility, for residents with household income less than 10,000 HK$ per month, the average walking time is around 4.87 min (SD ±5.79min); for residents with household income between 10,000 HK$ and 20,000 HK$ per month, the average walking time to the nearest PHC is around 4.68 min (SD ±5.06 min); for residents with household income between 20,000 HK$ and 40,000 HK$ per month, the average walking time is around 4.82 min (SD ±5.61 min); and for residents with household income over 40,000 HK$ per month, the average walking time is around 5.73 min (SD ±7.21min). People in the middle-income range spend less time accessing PHC facilities than people in the low- and high-income groups – while people in the highest income group spend the longest time to access PHC facility. With increasing income, the accessibility of PHC services increases and then becomes worse.

In terms of availability, for residents with household income less than 10,000 HK$ per month, the average Ai value is around 2.12 (SD ±1.42); for residents with household income between 10,000 HK$ and 20,000 HK$ per month, the average Ai value is stable around 2.12 (SD ±1.36); for residents with household income between 20,000 HK$ and 40,000 HK$ per month, the average Ai value is stable around 2.12 (SD ±1.40); and for residents with household income over 40,000 HK$ per month, the average Ai value decreases to 1.97 (SD ±1.48). Availability of PHC services is similar for low- and middle-income groups, while the high-income group has the poorest availability of PHC services. Thus, with increasing income, the availability of PHC stays constant and then becomes poorer.

## Discussion

### General discussion

With regards to the accessibility of PHC in Hong Kong, our results reveal that 95% of residents can access a PHC facility within 15 minutes, which is a good achievement from the perspective of geographic access time compared with other regions [27, 56, 69–72]. It indicates that the medical resources of PHC in Hong Kong, are fairly evenly distributed among residents such that most residents can access one PHC institute conveniently. It is noteworthy that the private medical institutions make a major contribution to the accessibility of PHC for Hong Kong residents, as the number of private PHC facility in Hong Kong is much greater than that of public PHC facilities. As the results reveal, only 56% of residents can reach a public PHC institution within 15 minutes, while 95% of residents can reach a private PHC institution within 15 minutes.

If PHC is accessible, is the supply of services sufficient to meet residents’ demand? Considering the capacity to provide primary health services, we found that 8% of residents can receive a public PHC service within the threshold range of 15 minutes’ walking distance in a month, while 67% of residents can receive a private PHC service. Combining the capacity of public and private PHC, 83% residents in Hong Kong can receive a PHC service. Thus, it is evident that private institutions have great advantages over public institutions in terms of capacity to meet residents' primary health care needs.

If PHC is available, then can people afford it? The public medical system in Hong Kong, including PHC services, is free and accessible to all residents. Due to the high cost of private medical systems, private medical services have a threshold for most residents, especially those who do not possess private medical insurance. In Hong Kong, people's medical insurance mainly covers hospital products, while the coverage rate of doctor consultation is low [64]. If uninsured residents seek public services and insured residents seek private services, the availability of primary medical services for Hong Kong residents will become less affordable. As the results indicated, only 12% of residents in Hong Kong can afford PHC service; while with the support of Medical Voucher, 32% residents in Hong Kong can afford PHC service. Even with the intervention of both medical insurance and medical vouchers, 68% of Hong Kong residents still cannot afford PHC.

The poorer accessibility among high-income groups, reflects that the distance between residential address and PHC facilities is longer compared with other groups, which means they have poorer access to PHC. But this kind of inconvenience is because we set walking as the way for residents to seek health care providers. High-income people have access to more modes of travel, and walking is not the main way they seek medical treatment. The use of walking as the main mode of travel for PHC accessibility in this paper therefore underestimates the actual accessibility and availability of PHC services, especially for high-income residents. What’s more, it’s noticeable that among these subgroups, the highest income group has the largest differences in accessibility and accessibility from the other groups, while the differences between the other groups are not particularly large. One possible explanation is that the truly wealthy people tend to live in the suburbs, which may make their physical distance to health care even longer with lower accessibility and availability to PHC on foot.

### Implications

During the outbreak of the COVID-19 pandemic, the PHC system has provided not only primary medical services, but also partial public health services. In Hong Kong, some of the primary medical institutions are entrusted by the government to provide vaccination services to the public and consultations and drugs to COVID-19 patients. Sound PHC systems can enhance regional public health response capacity, by making full use of its basic network of close ties with residents. Accessibility of PHC essentially reflects the spatial distribution of institutions, and the accessibility focuses on the spatial distribution of medical resources. Thus, a good accessibility and availability of PHC reflects that the network layout of existing PHC systems in Hong Kong is reasonable and can meet the needs of most residents.

The accessibility and availability of PHC in Hong Kong is good, but the affordability of PHC is not ideal. Low coverage of medical insurance in outpatient services has aggravated the crowding of public medical services and led to the underutilization of private PHC resources. It will be hard to realize the role of private institutions without addressing the issue of affordability of private services. Therefore, we suggest diverting patients to private institutions through medical insurance, medical vouchers, or other ways to relieve the pressure on the public system and make full use of the existing PHC network in Hong Kong. The government has been aware of the problem of crowded utilization of public medical resources and taken active measures to divert patients from public medical institutions to private institutions. In 2019, it launched a policy called the Voluntary Health Insurance Scheme (VHIS) [73]. The purpose of VHIS is to provide residents with an additional choice of using private healthcare services through hospital insurance and relieve the pressure on the public healthcare system. However, the scheme covers only hospital products. If primary health care products are included, it can also help alleviate crowding of primary health care in public institutions.

### Limitations

Our study possesses some limitations. First, we use walking as the mode of travel to seek PHC services, ignoring other possible options. This makes the results of accessibility in this paper pessimistic. However, the accessibility of primary health care services in Hong Kong is already good on foot, and if other transport factors are considered, the coverage of PHC will increase, and the time of access will be further reduced with additional costs. PHC is set for the whole residents, but the elderly are an important target group for primary health care. Moreover, the elderly experience restricted mobility. Thus, it is appropriate to take walking as the mode of travel to access primary health care. If precise geographic accessibility is required, other travel patterns may need to be added. Second, the number of visits accepted by public PHC institutions for a month was obtained from official reference data, but the number of visits accepted by private institutions was estimated. Assuming each private clinic has one registered doctor who works five days a week and treats 20 patients per day, then a private clinic can provide 400 visits per month. In calculating the availability, we set 400 visits as the monthly volume of services provided by each private clinic. A clinic may have more than one registered physician, so the availability of private primary health care services measured in this paper is underestimated. Even if the number of services provided by private clinics was underestimated, the accessibility of residents calculated in this paper was good, and the accessibility value would be even greater. Third, in calculating the affordability of PHC services, we assumed that people without medical insurance prefer public services, while people with medical insurance prefer private services. Moreover, all residents aged over 65 were assumed to be eligible for medical vouchers and to prefer private services in seeking PHC. Other factors affecting health behavior like the severity of the disease and economic status were ignored in this paper. Finally, in analyzing potential factors impacting the accessibility, availability, and affordability, the smallest geographic unit used in this paper is the LSBGs, and variables, such as age, education, income status, were all descripting characteristics of LSBGs. If we could get more detailed individual data in future, we can further analyze a more completed demographic factor analysis, including gender, marriage status, career and so on. What’s more, this paper aims to provide a general view of accessibility, availability, and affordability of residents of PHC to residents in Hong Kong. In future, in-depth analysis is needed to research on the primary care services for vulnerable groups, such as the differently abled and critically ill patients.

## Conclusion

Since the outbreak of COVID-19, the importance of primary health care networks has come to the fore. Providing not only primary medical services, they can also serve as a grassroots network for public health. For government, it is easy to redeploy medical resources from public medical institutions, so public health services are basically carried out in public medical institutions. In general, such an arrangement is feasible, but in public health emergency situations, it is not enough, and the government must clearly recognize the importance of the private sector in primary health care providing and understand and utilize the entire resource of the PHC system, including the private sector, to a achieve maximum efficient coverage of all residents.

In Hong Kong, 95% of residents can reach a PHC institution within 15 minutes and 83% residents can receive a PHC service within 15 mins walking in a month. Most people can reach an institution in a short time, validating showing the rationality of the spatial distribution of primary health care facilities. Most people can receive the PHC service they need within a month, showing the extensive and sufficient allocation of medical resource of the PHC network in Hong Kong. Moreover, the private sector holds a dominant position in Hong Kong’s PHC system, both in terms of accessibility and availability. As our results show, 56% of residents can reach one public PHC institution within 15 minutes, while 95% of residents can reach one private PHC institution within 15 minutes. Furthermore, 8% of residents can receive public PHC services within the threshold range of 15 minutes’ walking distance in a month, while 67% of residents can receive private PHC services.

According to the results of our regression analysis, there is no significant evidence that the elderly, native residents, low-educated population, nor owner-occupier households are significant influencing factors. However, post-secondary education and household income show significant impact on the accessibility and availability of PHC. Regions with higher concentrations of residents with post-secondary education attract greater targeting of PHC resources; while regions with higher concentrations of high-income households exhibit poorer accessibility and availability of PHC. Middle-income groups spend the least time to reach a PHC facility compared to low- and high-income groups, while the highest income group spend the most time. Thus, the availability of PHC services is similar for low- and middle-income groups, while the high-income group experiences the poorest availability of PHC services. No serious difference between social groups in accessibility and availability, further shows the equality in resource allocation of PHC services in Hong Kong.

However, there is a large gap between the availability and affordability of PHC in Hong Kong. In terms of availability of PHC, 83% residents in Hong Kong can receive PHC services in a month. By contrast, in terms of affordability, only 32% residents in Hong Kong can afford these services with the support of medical insurance and medical voucher. Narrowing the gap between availability and affordability is the critical for fully utilizing the capacity of the primary health care system in Hong Kong. Low coverage of medical insurance in outpatient services aggravates the crowding of public medical services and leads to the underutilization of private PH|C. Thus, we suggest diverting patients to private institutions through medical insurance, medical vouchers, or other ways, to relieve the pressure on the public system and make full use of the existing primary health care network in Hong Kong.

## Data Availability

The datasets used and analysed during the current study available from the corresponding author on reasonable request.
